# Current Treatments of Post-traumatic Stress Disorder and Amygdala Ablation as a Potential Cutting-Edge Therapy in Its Refractory Cases

**DOI:** 10.7759/cureus.31943

**Published:** 2022-11-27

**Authors:** Priyadarshi Prajjwal, Pugazhendi Inban, Balaganesh Natarajan, Spandana Mitra, Tamara Tango, Aneeqa Ahmed, Soniya Bansode, Abdullah A Almushawah

**Affiliations:** 1 Neurology, Bharati Vidyapeeth (Deemed to be University) Medical College, Pune, IND; 2 General Medicine, Government Medical College, Omandurar Government Estate, Chennai, IND; 3 Neurology, St. George's University School of Medicine, True Blue, GRD; 4 General Medicine, Employee's State Insurance Corporation (ESIC) Medical College & Hospital, Kolkata, IND; 5 Neurology/Neurosurgery/Internal Medicine, Faculty of Medicine, Universitas Indonesia, Jakarta, IDN; 6 General Medicine, Shadan Institute of Medical Sciences, Peeramcheru, IND; 7 Neurology, Government Medical College, Siddipet, Siddipet, IND; 8 Neurology, King Saud University, Riyadh, SAU

**Keywords:** combat fatigue, shell shock, cognitive behavioral therapy, prolonged exposure, trauma, post traumatic stress disorder, amygdala ablation

## Abstract

Post-traumatic stress disorder (PTSD)is a very common psychiatric disorder occurring in an individual of any age, gender, or race who underwent trauma, with women being twice more at risk than men. It is generally seen more in American Indians, United States Latinos, and African American ethnic groups. A patient is diagnosed with PTSD if the symptoms of intrusion, avoidance, changes in cognition and emotions, arousal, and mood reactivity changes persist for more than a month and cause the individual severe difficulty in their everyday cognitive and psychological functioning. The psychological treatment includes numerous therapies including trauma-focused therapies such as cognitive behavioral therapy, cognitive processing therapy, prolonged exposure therapy, eye movement desensitization and reprocessing, and non-trauma-focused therapies such as relaxation techniques, interpersonal therapy, and mindfulness. Various pharmacological measures have also been tried with mixed results such as selective serotonin reuptake inhibitors, benzodiazepines, adrenergic drugs, atypical antipsychotics, and mood stabilizers like lithium and valproate. As numerous studies have proven, PTSD is linked with right-side stimulation of the amygdala. The purpose of this article is to highlight the use of extremely selective laser ablation of the amygdala-hippocampal unit as a successful surgical intervention for medically unresponsive PTSD and as a revolutionary solution and prospective cutting-edge therapy in the near future.

## Introduction and background

Post-traumatic stress disorder (PTSD) is a common psychiatric condition in which an individual may encounter or experience a terrible or horrifying occurrence where there was substantial bodily harm or injury, which creates a sense of disturbance in his/her thought process. PTSD can result from traumatic events that leave victims feeling horribly afraid, helpless, frightened, or terrified. Sexual, emotional, or physical abuse, an unanticipated loss of a family member, an accident, a battle, any crisis, or a catastrophic event are a few examples of scenarios that might cause PTSD [1]. Throughout history, PTSD has gone by a variety of labels, including shell shock during World War I and combat fatigue following World War II. It is very crucial to take into account that it does not only affect soldiers, militants, and combat personnel, but any person, of any background, race, culture, country, and age, may experience PTSD and its symptoms [2]. Women are twice as likely as men to get PTSD. American Indians, United States Latinos, and African Americans are the three ethnic groups most afflicted and have a greater likelihood of PTSD than the Non-Latino White population. One out of every 11 individuals may at some point in their lives be given a diagnosis of PTSD, which affects approximately 3.5% of American adults annually. A loud disturbing sound or even an unintentional touch can trigger significant violent emotions in those with PTSD, leading them to resist or push away circumstances or someone who reminds them of the distressing occurrence. Years after the horrific incident has passed, affected individuals may continue to endure profound, unsettling sensations and emotions pertaining to their traumatizing encounter. Nightmares, bad trips, or flashbacks may cause them to replay the incident in their mind several times. They might feel depressed, afraid, or angry, and they could also feel cut off from other people including themselves sometimes [3].

For PTSD to be recognized and diagnosed, a stressful traumatic event must be exposed to a person repeatedly. The engagement might also be indirect rather than direct. For instance, someone may get PTSD as a result of hearing of the sudden death of a close cousin or friend. It may also happen as a result of repeated exposure to horrifying, traumatic facts, such as when healthcare individuals are exposed to victims of child abuse or sexual abuse that may trigger memories of a similar incident from their early years. Numerous studies have proved that PTSD is linked to right-side amygdala activation. In contrast to conventional surgical intervention, extremely selective laser ablation of the amygdalohippocampal unit is a successful surgical intervention for medically unresponsive medial temporal lobe epileptic seizures. 

Methods

**Figure 1:**
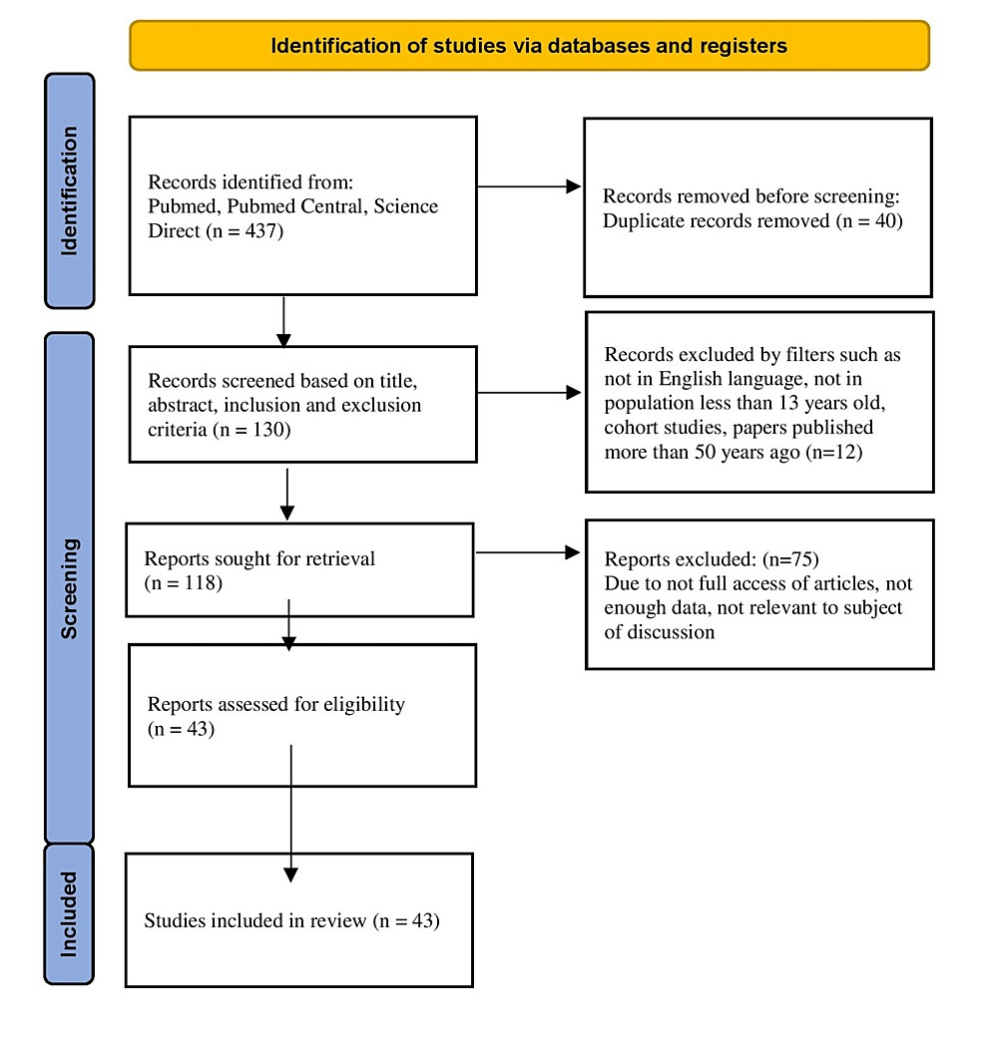
PRISMA Flow Diagram PRISMA: Preferred Reporting Items for Systematic Reviews and Meta-Analysis

Preferred Reporting Items for Systematic Reviews and Meta-Analysis (PRISMA) guidelines were implemented to achieve a smooth, seamless, and systematic review experience (Figure 1).

Search Strategy and Selection Criteria

For this comprehensive systematic review, the PubMed Central, PubMed, and Science Direct libraries were searched for articles published between January 1992 and January 2022 using the search terms “Amygdala Ablation”, “Post-traumatic Stress Disorder”, “Trauma”, “Prolonged Exposure”, “Cognitive Behavioral Therapy”, “Shell Shock”, and “Combat Fatigue”. The inclusion and exclusion criteria are given in Table 1.

**Table 1:** Inclusion and Exclusion Criteria

Inclusion Criteria	Exclusion Criteria
Studies focusing on teenagers, adults, and geriatric populations	Studies in populations aged less than 13 years old
Published systematic reviews, case studies, randomized controlled trials, meta-analyses, animal experiments, and literature reviews.	Cohort studies
Papers written in the English language	Papers not written in the English language.
Papers published during 1992-2022	Papers published earlier than 1992
Papers relevant to the subject of discussion.	Papers irrelevant to the subject of discussion.

## Review

Diagnosis of PTSD

PTSD symptoms can vary in severity from individual to individual. The five major categories that can be used to group PTSD symptoms are given below.

Intrusion

Types of intrusive emotions include recurrent, unwanted ideas, disturbing nightmares, or flashbacks of the terrible incident. Flashbacks are frequently so powerful that victims may think they are reliving the awful incident or seeing it directly before their eyes.

Avoidance

Victims of PTSD usually stay away from people, objects, hobbies, and circumstances that can trigger painful memories. One could try to resist remembering or worrying about the unpleasant experience. People can be unwilling to talk about what happened or even how they are feeling because of what had transpired.

Cognitive Changes

The failure to remember crucial points of the stressful incident, negative thoughts leading to chronic distorted and persistent beliefs about oneself or others, altered ideas about the intent or ramifications of the event leading to wrongfully casting aspersions on oneself or others, a significant decline in interest in the once enjoyable activities, and a disengaged or estranged feeling are examples of modifications in cognition and emotions.

Changes in Cognition and Emotions

This includes the failure to recall crucial details of the stressful incident, negative emotions leading to persistent and distorted beliefs about oneself or others, distorted ideas about the purpose or repercussions of the event leading to incorrectly placing blame on oneself or others, far less interest in once-enjoyed activities, and a detached or estranged feeling (a void of happiness or satisfaction).

Arousal and Mood Reactivity Changes

Arousal and reactive indicators can include irritability and violent outbursts, risky or self-destructive behavior, being suspiciously highly aware of one's surroundings, being quickly startled, having trouble focusing, or having trouble sleeping. This can also include anger and violent eruptions, dangerous or self-destructive actions, being very acutely aware of one's surroundings, being jolted suddenly, having difficulties concentrating, or experiencing problems sleeping.

**Figure 2:**
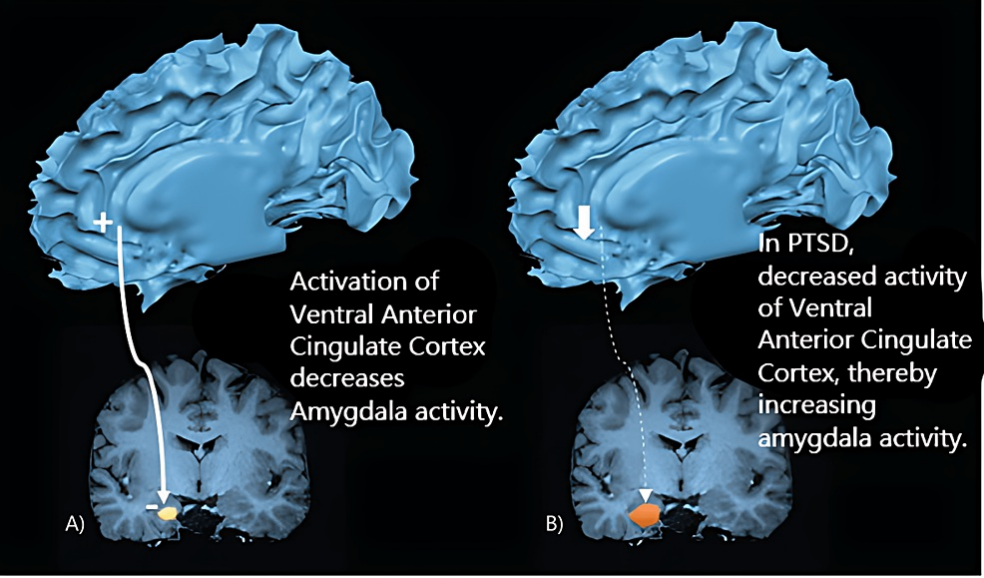
Role of Amygdala in PTSD: (A) Stimulation of Ventral Anterior Cingulate Cortex decreases Amygdala Activity, (B) Inhibition of Ventral Anterior Cingulate Cortex, as in PTSD, increases Amygdala Activity PTSD: Post-Traumatic Stress Disorder Image modified from: Forster et al., 2017 [4]

Many people experience symptoms mimicking those listed above in the weeks that followed a stressful event. The symptoms of PTSD typically occur within three months after the stressful incident, but they can occasionally take many months or years to manifest. Over the period of many years, symptoms may appear and disappear. In PTSD, the symptoms usually occur due to the decreased activity of the ventral anterior cingulate cortex, which results in increased amygdala activity (Figure 2). An individual must experience at least one re-experiencing complaint, three avoidance symptoms, two detrimental changes in mood or cognition, as well as at least two of the hyperarousal symptoms for a minimum of one month in order to be diagnosed with PTSD. The individual's capacity to carry out daily tasks must be hampered by these symptoms. People who have the disease may also misuse alcohol or drugs. Additionally, they could feel pain, stress, anxiety, depression, palpitations, tachycardia, and perspiration. The symptoms of anxiety in adolescents and children may be varied from those in adults. Bedwetting, communication and language problems, acting out the incident during playing, or being particularly clingy are all indicators that can occur in very early childhood.

Current recommended treatments

There are numerous psychological therapies addressing PTSD, including both trauma-focused as well as non-trauma-focused therapies. Treatments that are trauma-focused deal specifically with memories and recollections of the traumatic incident or with feelings connected to the horrific situation [5]. For instance, both cognitive behavioral and processing therapy, and prolonged exposure represent trauma-focused therapies. Interventions that are not trauma-focused try to lessen behavioral problems without specifically addressing the distressing event's flashbacks, emotions, or sensations. Relaxation, mindfulness, stress inoculation training (SIT), and interpersonal therapy are some non-trauma-focused therapies [6]. There have been certain specific modalities tried for combat-related PTSD too [7], which included focussed behavioral therapy as well as the use of antidepressant medications such as mirtazapine in some cases [8]. Some of the widely accepted current therapies are given below.

Cognitive Processing Therapy (CPT)

According to CPT, after a traumatic incident, victims try to comprehend what had actually happened, which frequently results in skewed beliefs about themselves, their thoughts, the outside world, and other people [9]. Individuals frequently integrate, acclimatize, assimilate, blame themselves, blame their fortunes, and over-accommodate in an effort to reconcile the tragic experience with previous schemas making them believe it might happen to them again [10]. Assimilation is the process by which new facts are modified to support their pre-existing ideas, and beliefs which can lead to a self-blame for the distressing occurrence. Over-accommodation is the act of altering one’s views in an attempt to stop trauma from happening again. This can lead to ideas that the world is unsafe or that people are not dependable [11]. CPT identifies these maladaptive thoughts that have developed as a result of the traumatic experience while allowing cognitive stimulation of the memory. According to Resick and Schnicke [12], CPT’s primary goal is to change the perception of traumatized individuals to promote accommodation. The main goal of treatment is to assist the patient in reinterpreting and rethinking their horrific experience in order to lessen the lasting detrimental consequences it has on their present state of affairs. This requires the client to explore, examine, assess, and reaccess their meta-emotions and altered thoughts caused by the event, which cannot be done without lowering the fear of the event in the person's mind.

Understanding the background of PTSD, its thought mechanisms, and the emotions behind it is the initial stage. By developing a shared understanding of the patient's issues and describing the psychological approach to PTSD creation, the therapist aims to create a rapport with the patient and win their cooperation. To build a realistic foundation of the patient's perspective of why the incident occurred and the influence that it has exerted on their views about self, society, and the world [12], the psychiatrist requires the client to produce an assessment report. Finding thought patterns and creating awareness of the connection between such a person's feelings and thinking are the main goals of this phase. Helping the client to acknowledge unhelpful ideas (also known as "stuck points") that obstruct their capacity to recover from tragic incidents is a particular area of emphasis.

The formal processing of the trauma comes second. The patient is asked to write a thorough description of their greatest traumatic incident, which they then recite aloud to the therapist during a session. With the overall goal aiming for the individual to examine and adjust their maladaptive cognitions, this is meant to interrupt the cycle of avoidance and promote interpersonal interactions. Since the patient's own discovery of new thinking patterns regarding their trauma, as contrasted to uncritical acceptance of the clinician's views, is crucial to healing, therapists frequently utilize Socratic questioning to discreetly prod the client [12]. Alternative CPT procedures include CPT-cognitive, which some doctors have considered to be equally beneficial, if not more so, than CPT using written reports.

In order to put those abilities to further recognize, assess, and adjust their beliefs about their traumatic events, the client is assisted in reinforcing the techniques they acquired in the prior phase of treatment during the last phase of treatment. The five conceptual domains of safety, esteem, control, trust, and intimacy that traumatic events most frequently harm are the emphasis of this phase. The goal is for the patients to leave therapy feeling capable and confident enough to apply appropriate adaptive coping mechanisms in their post-treatment lifestyles. Clients work on understanding how their traumatic events led to overly broad maladaptive ideas and how those beliefs affect their present performance and life quality.

Prolonged Exposure Therapy

Prolonged exposure therapy is a useful first-line treatment for PTSD, regardless of the cause [13]. It has been specifically advocated for use by military members and survivors, depending on the sort of trauma. This view is supported by a large number of research and clinical practice standards and guidelines from various organizations. Prolonged exposure works well in lowering PTSD signs and symptoms as well as has shown effectiveness in lowering comorbid associated difficulties with PTSD such as apathy, negative emotions, remorse, blame, despondency, and health views. Prolonged exposure has been effective in groups with complex diagnostic issues and evacuees of tragedies caused by a single incident or multi-level trauma [14]. The standard protocol for prolonged exposure contains four primary therapeutic elements (i.e., psychoeducation, in vivo exposure, imaginal exposure, and emotional processing). Given the effectiveness of prolonged exposure, the Veterans Health Administration created a prolonged exposure training program. The program serves as an important one for prolonged exposure therapy advocacy by training over 1300 service providers in the mental health field.

Eight to 15 separate 90-minute sessions make up the current prolonged exposure program for the therapy of PTSD. The clinician gives a thorough justification for exposure therapy in the initial consultation and outlines the two main issues that keep PTSD from getting better [15]. The first step is avoiding flashbacks of the trauma as well as impulses and pictures connected to it. Though suppression is successful in lowering anxiety in the short term, therapists add that it sustains PTSD by eliminating opportunities to analyze as well as integrate the trauma experience psychologically. The second element is the unwanted and frequently incorrect beliefs that have emerged as a result of the trauma. For instance, many trauma survivors have the erroneous notion that the environment is really hazardous and that they are totally incapable of coping with it. As a result, prolonged exposure attempts to change misconceptions by giving the victims the chance to learn through experiencing opportunities [16] that contradict these false beliefs (i.e., exposure).

The doctor and patient must decide which trauma to emphasize during imaginal exposure during the first session. This "index trauma" is chosen for individuals with a history of several traumas by identifying the incident that is actually leading to the most discomfort and dysfunction. This is frequently the incident tied to the distressingly frequent recurrence of symptoms [17]. In either the primary patient assessment or the first session, the index trauma is chosen as part of the trauma history questionnaire. In the session, patients are also taught a slow-breathing relaxing method, which they are urged to use daily as an assignment.

*Cognitive Behavioral Therapy *(*CBT)*

Depending on behavioral and cognitive frameworks, trauma-focused CBT frequently incorporates elements of prolonged exposure and CPT as well as other CBT theories. For instance, Ehlers and Clark suggested that sufferers with PTSD have overly pessimistic views of the trauma and also their memories of the trauma are marked by sluggish contextualization, powerful causal memories, and powerful perceptual induction, which causes uncontrollable reliving of the trauma again and again [18]. According to Kubany et al., guilt-associated assessments may arouse negative emotions and be accompanied by memories or recollections of the trauma [19]. The continuous conditioning of trauma memories with suffering by these guilt assessments may result in impulses to repress or avoid stimuli associated with the event. According to Ehlers and Clark, people with PTSD employ dysfunctional behavioral and cognitive coping mechanisms which keep patients from altering their unfavorable self-perceptions and traumatic memories [18]. As a result, this therapy aims to alter negative judgments, restore memory performance, and get rid of undesirable behavioral and cognitive patterns. 

Trauma-focused CBT frequently combines behavioral strategies like exposure with cognitive strategies like cognitive restructuring. Putting the painful narration down or reading the terrible memory aloud are examples of CBT techniques that employ exposure to the traumatic experience [19]. In vivo exposure is frequently used in CBT because it involves exposition to the trauma-related stimuli, or patients are taught how to recognize relapse triggers and practice discriminating between "then vs. now" [20]. The goal of cognitive reconstruction is to ensure that patients recognize dysfunctional thoughts and cognitive biases, elicit reasonable alternative concepts, and reevaluate their views about themselves, their trauma, and the outside world [21].

Eye Movement Desensitization and Reprocessing (EMDR)

Shapiro introduced EMDR therapy, a type of psychotherapy that is frequently employed in treating PTSD sufferers. In EMDR, the subject imagines being exposed to the trauma whilst making saccadic eye motions. In order to retrieve painful memories as well as receive a bilateral stimulus, patients must split their attention and focus [22]. There have been numerous theories put out to explain how ocular movement in EMDR functions. The working memory hypothesis of the EMDR functionality has garnered the most empirical evidence; it states that when recalling painful memories if the eye movements are done concurrently, the working memory will become less effective since ocular movements consume multiple processes in the brain [23]. This could cause the trauma picture to lose strength even after being reconsolidated into episodic memory. Therefore, it is believed that the function of ocular movements in EMDR is to make painful memories less distinct and traumatic, leading to overall enhancements and improvements in post-traumatic symptoms [24].

Deep Brain Stimulation (DBS)

Around 30-50% of PTSD patients do not respond to gold-standard treatment such as the trauma-focused therapies discussed above. Other alternatives to manage refractory PTSD have been reported, such as DBS. Electrodes are placed in the basolateral nucleus of the amygdala of PTSD patients and are stimulated intraoperatively by high-frequency DBS [25]. A study by Langevin et al. showed that PTSD patients had decreased frequency of nightmares following DBS [26]. Several animal studies indicate that activation of the amygdala, hippocampus, ventral striatum, or prefrontal cortex could be useful in the suppression of anxiety and stress behavior in PTSD, however, the use of DBS for PTSD is currently purely experimental. The sparse human data are consistent with the potential security and efficacy of high-frequency DBS of the basolateral amygdala (BLA) in treating PTSD but still many more clinical trials are needed to support this hypothesis.

Pharmacological Treatment

There are many drugs that have been tried to varying success to effectively treat PTSD. These include monoamine oxidase inhibitors, serotonin reuptake inhibitors, and tricyclic antidepressants. Selective serotonin reuptake inhibitors stand out as the recommended first-line treatment option for PTSD when taking into account documented overall efficacy and side effect profiles. Mood stabilizers, atypical antipsychotics, adrenergic medications, as well as newer antidepressants such as mirtazapine also exhibit promise, but more controlled studies are needed to determine where they fit into the PTSD drug repertoire. Regarding the role of medication in the management of PTSD, there is a huge clinical debate. While some guidelines view medication as a first-line therapeutic approach, others advise turning to psychotherapy before pharmacological management of any kind. There have been several studies highlighting the role of prazosin in the management of PTSD nightmares but the current recommendations in this approach vary. While a more recent big Veterans Affairs Cooperative Study with negative outcomes had inconsistent findings about the efficacy of the drug [27], prior guidelines that were based on smaller favorable trials advocated the use of prazosin. In patients who respond well to the drug, prazosin is still utilized in therapeutic settings. Another hot prescription given by medical practitioners for PTSD is benzodiazepines. Even though close to 74% of patients who have been diagnosed with PTSD continue to be given medication of benzodiazepines, they are still not advised for the treatment of PTSD. In fact, benzodiazepines may hinder the release of fear conditioning, enhance the development of the response to fear, and exacerbate trauma recovery. Before using it to treat PTSD or stress related to PTSD, careful thought should be taken.

Amygdala ablation: a revolutionary solution in PTSD

Very little is understood about the neuronal networks that govern fear implantation as well as development in the complex physiological memory traces of the human brain. The central, as well as, basolateral nuclei of the amygdala are important parts of the neural pathways of both inherent [28] and acquired stress [29]. Hyperactivity of the amygdala occurs due to a deficiency of top-down modulation by the prefrontal cortex [30]. Besides that, increased top-down excitation from the amygdala to mPFC also contributes to amygdala hyperactivity. This idea is supported by a study conducted by Chen et al. where there was an increase in connectivity from the amygdala to the supplementary motor area and the amygdala to the precuneus [31]. As a result of amygdala hyperactivity, PTSD patients may experience hypervigilance and increased fear responses [32]. Moreover, PTSD also causes structural changes in the amygdala. Patients with PTSD were reported to have reduced volume of the amygdala. However, the reduction of amygdala volume was not associated with the severity of PTSD symptoms [33].

Considering how well the amygdala influences how we remember our fears, it seems likely that it is implicated quite actively in the pathogenesis of PTSD. Recent research and studies indicate that perhaps the incubation of substance cravings, which refers to a time-dependent rise in drug-seeking behavior following withdrawal, is a process that may be associated with the central amygdala nucleus [34]. Both the medial amygdala and cephalic regions of the amygdala have relatively high PTH2R activity and inputs through TIP39 neurons [35]. Situational fear reinforcing triggers the activation of neurons in the medial amygdala; therefore, it can be concluded that the lesions of the medial amygdala could and possibly do affect various fear behaviors, such as predator odor-evoked freeze response, fear-potentiated startling response, acute neuroendocrine bodily reactions, and reinforced fear memory, which are quite commonly seen in patients with PTSD.

Neuronal endings in the subparafascicular region generate medial amygdala TIP39 [36]. According to Wang et al., the subparafascicular area receives input from a variety of sensory as well as integrative regions of the brain, making it logical that it affects the condition of the medial amygdala and modifies the effects of traumatic experiences [37]. TIP39 apparently functions as a neuropeptide to affect the activity of medial amygdala pathways that interpret information from footshocks. There is also evidence that neuromodulators have significant impacts on state-dependent memories, specifically in fear conditioning [38]. 

Amygdalotomy is an invasive surgery that removes the unilateral and/or bilateral amygdala. This procedure can also be accompanied by hippocampectomy. Removal of the unilateral amygdala is not protective against PTSD. A study by Adami et al. reported a 20-year-old woman who developed PTSD symptoms two weeks following left amygdala hippocampectomy to treat her refractory seizures [39]. Another study by Smith et al. also showed the development of PTSD symptoms in a refractory epilepsy patient two years after undergoing left amygdalotomy [40]. Lastly, a study by Yrondi et al. demonstrated the occurrence of PTSD symptoms one month after temporal lobe epilepsy surgery in a 41-year-old male [41].

Amygdala ablation is an MRI-guided laser interstitial thermal therapy (LITT) that targets the amygdala. The use of LITT in the neurosurgical field has been observed in treating epilepsy, glioma, and brain metastases. Before the LITT procedure, stereotactic MRI imaging is performed intraoperatively to determine the point where the laser tip is inserted, the target area in the brain, and the trajectory angle. After the burr hole is created, a stereotactic bolt is placed. The number of the stereotactic bolt depends on the number of trajectories. Then the laser probe is inserted into the target area. Real-time color-coded thermal imaging is obtained during ablation to compare the extent of damage to the brain area before and after the procedures [42]. Amygdala ablation in PTSD patients was reported for the first time by Bijanki et al. in 2020 [43]. They reported two cases of amygdala ablations in PTSD patients who also had seizures. The first patient was a 62-year-old Caucasian male, a veteran of the Vietnam war, who had chronic refractory PTSD for 30 years and seizures for 14 years. He also did not tolerate CBT. The second patient was a 42-year-old African American woman who experienced multiple violence against family members for 19 years and seizures for three years. According to functional MRI results, both patients were left language dominant. Both patients underwent the same procedure namely right laser amygdalohippocampectomy to treat their middle temporal lobe epilepsy. The first patient remained seizure free during one year of follow-up. He also showed some improvements in complex attention, naming the famous person, and verbal memory. However, there was a slight decline in visual memory and pattern recognition. The second patient had decreased frequency and severity of seizures. One year after the surgery, she experienced decreased hyperarousal and negative alterations in cognition and mood, as well as improved memory and quality of life [43].

In the study conducted by Bijanki et al., unilateral amygdala ablation instead of bilateral amygdala ablation was performed on PTSD patients [43]. The underlying reasons may be explained by the fact that the presence of two amygdalae may assist in the development of PTSD symptoms [44], while the complete removal of the amygdala may lead to poor decision-making and disturbed emotional memories [45]. Having a non-dominant amygdala only may also cause the development of PTSD symptoms. Therefore, the target area of amygdala ablation focused on the non-dominant amygdala [46]. This finding is in line with Bijanki et al., which involved patients with a non-dominant amygdala on the right side [43]. Other than amygdala dominance, the side of the amygdala with increased activity should be taken into account. Several pieces of evidence demonstrated hyperactivity of the right amygdala in PTSD patients [47]. This may indicate the role of the right amygdala in developing PTSD symptoms. It also explains the finding of PTSD symptoms in a patient following the removal of the left amygdala.

Compared to open surgery, amygdala ablation is less invasive. The possibility of collateral damage can be minimized due to its superior precision [43]. Limited evidence of the successfulness of amygdala ablation among PTSD patients remains the challenge in implementing this alternative. The efficacy of amygdala ablation in treating PTSD symptoms only is still questionable as prior research showed that amygdala ablation is intended to treat epilepsy in patients with both epilepsy and refractory PTSD. Therefore, future studies should explore the utilization of amygdala ablation for refractory PTSD treatments.

Limitations of the study

There have been a few limitations in our comprehensive study, which should be mentioned and documented. There is a lack of a significant number of research, published clinical trials, and enough evidence highlighting the amygdala ablation intervention in the treatment of PTSD. The magnitude of our previous statement can be understood by the fact that amygdala ablation in PTSD was reported for the first time in 2020 by Bijanki et al. [43], and very few studies have been published on it to date. Many studies on it are still going on and it’s a quite modern approach so documentation on this is really quite less. Free full-text access was another important consideration when deciding which studies to use. Last but not least, due to the growing global interest in the topic of this study, there is indeed a risk of information bias.

## Conclusions

Both therapeutic and diagnostic challenges are frequently presented by PTSD. A thorough mental state assessment, cognitive testings, and lab tests are necessary to exclude any concomitant psychiatric or medical illnesses. A thorough history of the client may reveal that perhaps the person has PTSD. Early diagnosis and prompt treatments are crucial and require multidisciplinary cooperation. A variety of treatment recommendations are now available to support medical practitioners in treating patients with PTSD. Out of the number of trauma and non-trauma focussed therapies, which have existed for decades and been followed widely, such as CPT, prolonged exposure therapy, DBS, and pharmacological measures, to name a few, since the past couple of years an unconventional therapy has been becoming the cutting edge treatment for psychotherapy and medically refractory cases of PTSD, the amygdala ablation surgery. A huge number of research and trials are still required to prove the actual benefits against unwanted effects of this surgery in PTSD, but currently whatever the limited shreds of evidence suggest, amygdala ablation can be a revolutionary measure for those patients who have failed to respond to any conventional therapy and provide them a better quality of life.
